# Assessment of Nutritional Status and Its Influence on Ovarian Reserve: A Systematic Review

**DOI:** 10.3390/nu15102280

**Published:** 2023-05-12

**Authors:** Laura Prieto-Huecas, Clara Ángela Piera-Jordán, Verónica Serrano De La Cruz-Delgado, Ana Zaragoza-Martí, María Belén García-Velert, Cristina Tordera-Terrades, Miriam Sánchez-Sansegundo, Laura Martín-Manchado

**Affiliations:** 1 Obstetrics and Gynaecology Service, Hospital Marina Salud, 03700 Denia, Spain; lauraprieto94@gmail.com (L.P.-H.); claraangela.piera@marinasalud.es (C.Á.P.-J.); veronica.serrano@marinasalud.es (V.S.D.L.C.-D.); mariabelen.garcia@marinasalud.es (M.B.G.-V.); cristina.tordera@marinasalud.es (C.T.-T.); 2 Department of Nursing, University of Alicante, 03690 Alicante, Spain; lauramartinmanchado@gmail.com; 3 Alicante Institute for Health and Biomedical Research (ISABIAL-FISABIO Foundation), 03010 Alicante, Spain; 4 Department of Health Psychology, University of Alicante, 03690 Alicante, Spain; miriam.sanchez@ua.es

**Keywords:** ovarian reserve, fertility, nutritional status, body mass index, anti-Müllerian hormone

## Abstract

Background: Nowadays, there is a growing interest in the relationship among lifestyle, reproductive health, and fertility. Recent investigations highlight the influence of environmental and lifestyle factors such as stress, diet, and nutritional status on reproductive health. The aim of this review was to determine the influence of nutritional status on ovarian reserve in order to improve the reproductive health of women of childbearing age. Methods: A systematic literature review was carried out following the PRISMA method. The quality of the studies was assessed using the Cochrane Collaboration Risk of Bias tool. Data were extracted, and the results were summarized into two blocks: according to the technique used to assess ovarian reserve and nutritional status; according to the results found in the relationship between ovarian reserve and nutritional status. Results: A total of 22 articles involving 5929 women were included. In 12 of the included articles (54.5%), a relationship between nutritional status and ovarian reserve was demonstrated. In seven publications (31.8%), the increased body mass index (BMI) led to a decrease in ovarian reserve, two of them (0.9%) in patients with polycystic ovary syndrome, showing a decrease only if BMI > 25. In two articles (0.9%), there was a negative relationship between ovarian reserve and waist-to-hip ratio, and in one (0.45%), a positive relationship was shown between ovarian reserve and testosterone levels, the latter being related to body mass index. In five articles (22.7%), body mass index was used as a confounder and was negatively related to ovarian reserve, and in another four (18%), no correlation was found. Conclusions: Ovarian reserve appears to be influenced by nutritional status. A high body mass index has a negative impact on the ovary, decreasing antral follicle count and anti-Müllerian hormone. Oocyte quality is compromised, increasing the rate of reproductive problems and the demand for assisted reproductive techniques. Further studies are needed to understand which dietary factors have the greatest effect on ovarian reserve in order to promote reproductive health.

## 1. Introduction

There is growing interest in the links between lifestyle, reproductive health, and fertility. Against a backdrop of social change, recent years have witnessed an increasing trend towards delayed motherhood, with many women choosing to wait until their 30s or 40s to have their first child. According to the National Statistics Institute, the average age of Spanish mothers in 2021 was 33.05 years [1], which was above the European average of 29.5 years [2]. The data collected since the start of this registry also attest to a growing trend towards delayed motherhood in Spain. Moreover, an increasing number of couples are being diagnosed with infertility or conditions that make it difficult to become pregnant [2].

The World Health Organization’s definition of infertility is a “disease of the male or female reproductive system defined by the failure to achieve a pregnancy after 12 months or more of regular unprotected sexual intercourse” [3]. Infertility affects between 15% and 20% of couples of reproductive age in developed countries. In Spain, one in six couples have fertility issues, which is reflected in the growing demand for assisted reproductive technology (ART) procedures in recent years [2,4]. Concerns about rising infertility rates have intensified research into the environmental causes of infertility, although most research to date has centered on the influence of male infertility and sperm-related issues [5]. The main causes of female infertility are anovulation, endometriosis, fallopian tube disorders, pelvic adhesions, uterine anomalies, and diminished ovarian reserve [6].

Ovarian reserve refers to the number and quality of oocytes and is an indicator of reproductive potential [7]. It is one of the most important factors for achieving natural pregnancy and a strong predictor of ART success. Ovarian reserve is inversely correlated with maternal chronological age, the main determinant of reproductive capacity and success. Reproductive aging is considered to accelerate after the age of 35 years [8]. The assessment of ovarian reserve is therefore an important step in both the evaluation and treatment of infertility. Markers of ovarian reserve include antral follicle count (AFC), assessed by transvaginal ultrasound, and serum levels of various biomarkers, such as anti-Müllerian hormone (AMH), follicle-stimulating hormone (FSH), luteinizing hormone (LH), and estradiol (E2) [6,8]. While these markers are easy to measure, few studies have investigated the causes of diminished ovarian reserve [9].

Well-known factors associated with reduced oocyte quantity and quality are genetic alterations [10]; gynecological conditions, such as endometriosis, tumors, infections, and ovarian surgery; and eating disorders, such as anorexia nervosa [7,9]. Recent research has also highlighted the potential effects of environmental and lifestyle factors, such as chronic stress, exposure to certain endocrine disruptors, diet, and nutritional status [10,11,12,13].

Nutritional status is assessed using a range of anthropometric parameters, including body mass index (BMI), body fat composition (% of fat), body perimeters, and waist circumference or waist-to-hip ratio. BMI has been used for decades to classify individuals as underweight, normal weight, overweight, or obese [14]. Obesity has been defined as the pandemic of the 21st century due to its global effects on morbidity, mortality, quality of life, and health expenditure [15]. In 2020, 61.4% of men and 46.1% of women in Spain were considered to be overweight or obese [1]. Overweight and obesity are linked to an increased prevalence of reproductive disorders and chronic diseases, such as cardiovascular disease and certain cancers [16,17].

Several studies have shown that a BMI outside the normal range affects female reproductive capacity. Obesity has been associated with menstrual disorders, anovulation, hirsutism, and higher miscarriage and infertility rates [17]. Impaired fertility in women with a high BMI is linked to changes in the hypothalamic–pituitary–ovarian (HPO) axis that induces endocrine and metabolic disorders, with adipose tissue acting as a key regulator [18]. In obese women, the upregulation of enzymes involved in androgen metabolism in adipose tissue can cause hyperandrogenism. The increased peripheral aromatization of androgens to estrogens, combined with the reduced production of sex-hormone-binding globulin (SHBG), exerts negative feedback on the HPO axis in obese women, inhibiting folliculogenesis [18].

Another physiological mechanism underlying the effects of obesity on reproductive capacity is the association between obesity and insulin resistance [19]. Obesity causes adipocyte hyperplasia and hypertrophy, resulting in a greater volume of adipose tissue. As an endocrine organ, adipose tissue secretes multiple proteins known as adipokines. The expansion of this tissue contributes to altered adipokine profiles, with a predominance of proinflammatory cytokines (mainly interleukin-6 and tumor necrosis factor-α), resulting in low-intensity chronic inflammation that can cause muscle and liver insulin resistance [19]. Obese women are thus prone to chronic low-grade inflammation and insulin resistance, both of which are associated with impaired reproductive function and late spontaneous abortion after ART [20,21].

Based on the evidence suggesting an important role for optimal nutritional status in fertility, we designed a systematic review to assess the influence of nutritional status on ovarian reserve in women of reproductive age. 

## 2. Materials and Methods

We conducted a systematic review following the PRISMA (Preferred Reporting Items for Systematic Reviews and Meta-Analyses) framework [22]. Study quality was assessed using the Cochrane Collaboration Risk of Bias tool [23] (Higgins, J. et al., 2011), which consists of seven items covering six domains of bias. Each item is classified as having a high, low, or unclear risk of bias. 

### 2.1. Data Sources

Electronic searches were carried out in the international databases Medline, ScienceDirect, and the Cochrane Library. Additional articles were identified by a hand search of the reference lists of identified articles. 

### 2.2. Search Strategy

The search strategy was designed to identify full-text, published articles and included MeSH (Medical Subject Heading) terms and the terms title and abstract. The following keywords, transformed into MeSH terms, were used: “ovarian reserve”, “anti-Müllerian hormone”, “nutritional status”, and “body mass index” combined with the Boolean operators AND and OR. The search strategy used in PubMed is shown in Table 1. 

### 2.3. Article Selection

Articles for full-text review were selected by screening the titles and abstracts of all publications yielded by the systematic search of Medline, ScienceDirect, and the Cochrane Library. The articles were independently reviewed by two authors, who checked the inclusion and exclusion criteria. The quality of each study was assessed by the same authors working separately using the Crombie criteria adapted by Petticrew and Roberts. Any discrepancies were resolved by a third author. For cross-sectional studies, the AXIS critical appraisal tool [24] was used to assess quality and risk of bias (Table 2). The quality of cohort and case–control studies was assessed using the Newcastle–Ottawa Scale [25] (Table 3). Randomized clinical trials were assessed using the PEDro tool [26] (Table 4).

The first and second authors (L.P.-H. and C.Á.P.-J.) independently scored each article, with discrepancies resolved by agreement with the third author (A.Z.-M.). Cohen’s kappa statistic (κ) was calculated to assess interrater reliability for risk of bias assessments. Assessment of blinding of participants or observers was not performed as all the studies were rated as high risk by both authors based on the overall items. Interrater reliability analyzed using Cohen’s κ yielded an intraclass correlation coefficient of 0.8.

### 2.4. Inclusion and Exclusion Criteria 

The inclusion criteria were (1) open-access articles with an abstract and full text, (2) articles written in English or Spanish, (3) articles published between 2011 and 2021, and (4) articles that included women aged between 18 and 46 years. The exclusion criteria were (I) protocols and articles unrelated to the topic; (II) review articles, systematic reviews, and meta-analyses; (III) conference proceedings; (IV) studies involving women undergoing ART procedures; and (V) studies of women with a serious disease or a condition that could diminish ovarian reserve. 

### 2.5. Data Extraction

The first author extracted all relevant data from the articles, namely, year of publication (2011–2021), study design and objectives, year of study conduct, sample size, mean participant age, country of origin, study results, and conclusions.

### 2.6. Synthesis of Results

The data extracted from the texts were grouped into two blocks to analyze, including (I) the variables used to assess ovarian reserve and nutritional status and (II) the association between ovarian reserve and nutritional status. 

## 3. Results

**Figure 1:**
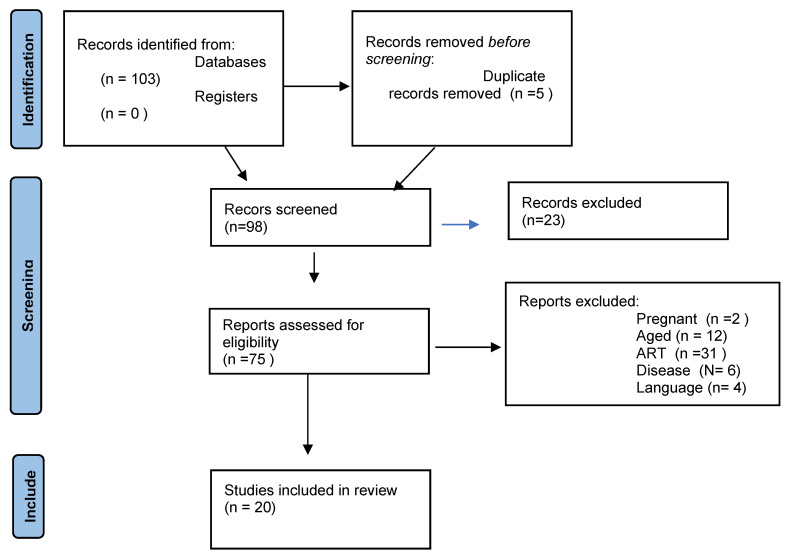
Selection of the studies.

The search yielded 103 articles. Five duplicate studies were removed before the selection process. Of the remaining 98 articles, 23 were excluded due to duplication and 55 due to exclusion criteria. Twenty studies were thus included in the systematic review (Figure 1).

### 3.1. Description of Study Characteristics

The characteristics of the articles included in this systematic review are summarized in Table 5. Six articles were conducted in the USA [28,29,30,31,32,38], two in France [42,44], two in China [34,43], and one each in Taiwan [21], Australia [33], Scotland [41], Turkey [45], the United Arab Emirates [40], Israel [46], India [35], Brazil [36], Denmark [37], and the Philippines [39]. The mean age of the study participants was 32.3 years. 

There were eight cross-sectional studies [27,28,29,30,31,32,33,34], seven cohort studies [35,36,37,38,39,40,41], four case–control studies [42,43,44,45], and one randomized clinical trial [46]. 

The total number of participants analyzed in the 20 studies was 5929.

### 3.2. Description of Study Variables

The study variables used to assess nutritional status and ovarian reserve are summarized in Table 6. BMI was used to assess nutritional status in all the studies. Five studies also measured weight and height [32,33,34,43,45], and one assessed waist circumference [31].

AMH was used exclusively to assess ovarian reserve in seven studies [30,31,32,33,38,39,46]. Six studies used a combination of AMH and other serum biomarkers, namely FSH and LH [34,37,41,42,43,45]. Eleven studies used serum markers and AFC assessed by transvaginal ultrasound [27,28,29,35,36,37,40,42,43,44,45].

Three studies evaluated biochemical parameters (cholesterol, high-density lipoprotein (HDL), and triglycerides) [31,32,41], and five included smoking as a confounder [28,29,30,37,38].

### 3.3. Relationship between BMI and Ovarian Reserve

Table 7 shows the relationship between BMI and AMH, together with the results and conclusions of each study. Five of the studies concluded that a high BMI was associated with diminished ovarian reserve based on below-normal serum AMH levels or AFC [27,28,35,41,44]. One of these studies [41], which divided the sample into three subgroups based on cardiometabolic, psychological, and reproductive profiles, concluded that low ovarian reserve was associated with cardiovascular and psychological factors. Another [29] found correlations between a high BMI and diminished ovarian reserve, particularly in Caucasian women. Finally, one study reported that a high BMI had a negative effect on inhibin B levels [35].

Two studies [31,32] indicated a possible positive relationship between a healthy cardiometabolic profile and AMH levels. Bleil et al., who investigated the association between variations in reproductive aging and cardiovascular risk factors, also determined that low and medium AMH levels were associated with a larger waist circumference and higher cholesterol, indicating the need for longitudinal studies to determine whether this association is mediated by BMI [31]. The same group, in a later study of ovarian reserve, reported that infertile women had a larger waist circumference than fertile women [28].

Two studies of women with polycystic ovarian syndrome (PCOS) reported differences in ovarian reserve between women with a high and a normal BMI [27,44]. Both detected significant differences and found that women with PCOS and a BMI > 25 had lower AMH levels than those with a BMI within the normal range. Yang et al. also investigated associations between iron levels, obesity, and ovarian reserve in women with PCOS, finding evidence of diminished ovarian reserve and higher iron levels. These variables correlated with insulin resistance and reduced menstrual period frequency.

Another study investigating the association between AMH and metabolic syndrome in women with PCOS concluded that AMH was positively correlated with HDL cholesterol and SHBG and negatively correlated with glucose, insulin, blood glucose, BMI, and blood pressure [32]. Table 8 shows a summary of the relationships between BMI, AMH, and AFC.

## 4. Discussion

The aim of this systematic review was to evaluate the association between nutritional status and ovarian reserve in women of reproductive age. Eleven of the twenty studies analyzed showed lower AMH levels in obese women [27,28,29,32,34,35,36,41,44]. Five studies included BMI as a confounder [30,33,40,43,46], and the other six found no evidence that nutritional status influenced ovarian reserve [31,38,39,40,41,46].

The above findings are consistent with previous reports. Vitek et al. studied the relationship between BMI, AMH, and oocyte yield in women undergoing in vitro fertilization; of the 29,895 women studied, 16,579 were obese, and 13,316 had a normal BMI. Compared to normal-weight women, women with a high BMI had lower AMH levels (1.8 ± 2.0 vs. 2.1 ± 2.0, *p* < 0.001) and a lower oocyte yield (11.9 ± 7.3 vs. 12.8 ± 7.7, *p* < 0.001) [47].

Several publications have reported a negative correlation between BMI and AMH in women of late reproductive age [48,49]. Freeman et al., in a study of 122 women with a mean age of 45.8 ± 5.2 years, found that AMH levels were 65% lower in obese women than in non-obese women [48]. Steiner et al., who investigated the effects of oral contraceptive use and AMH levels in obese and normal-weight women, observed a 34% reduction in AMH levels in the former [50]. Another study observed a correlation between obesity and biochemical and ultrasound markers of ovarian reserve, showing lower AMH levels in obese women compared to normal-weight women of a similar age. AFC, by contrast, was similar in both groups [51]. Marca et al. found that AMH levels decreased with increasing BMI [52].

Ovarian reserve can also be assessed by AFC. We observed lower oocyte quality and AFC in overweight and obese women, confirming previous findings [29,35,44,53,54,55]. Chronic inflammation caused by obesity induces ovarian oxidative stress, which affects the different stages of folliculogenesis (development, maturation, and ovulation). Some authors have indicated that obesity might affect oocyte quality via lipotoxicity, a mechanism marked by the persistent, unregulated release of cytokines from adipose tissue which have even been detected in follicular fluid [35,56,57]. Several authors have suggested that ART could improve the chances of pregnancy in obese women, albeit with an increased risk of spontaneous abortion [56,58] and implantation failure [54]. These risks could be reduced by using oocytes from lean donors [59,60].

This systematic review confirms previous findings showing that metabolic syndrome, understood as a combination of central obesity, elevated blood pressure, elevated triglycerides, elevated fasting glucose, and reduced HDL-cholesterol, has an important role in female fertility. Cardozo et al. suggested that metabolic syndrome might also affect endometrial receptivity as it appears to influence both oocyte and embryo quality [61]. Similarly, Snider et al. found that obesity-dependent changes in the gut microbiome contributed to lower oocyte quality [57].

Several studies have reported contrasting findings on the link between nutritional status and ovarian reserve [39,53,62,63,64,65], possibly because of the use of small samples with few obese women. Dolleman et al., for instance, in a study of 2320 women of reproductive age, found that AMH levels were lower in oral contraceptive users and did not correlate with BMI [53]. Sahmay et al. suggested that reduced fertility rates in obese women are more likely to be due to impaired endometrial receptivity [62].

This systematic review has some limitations. First, we may have missed some evidence as we only analyzed studies published in English and Spanish. Second, we searched just three databases: Medline, Science Direct, and the Cochrane Library. Future studies could target additional databases such as Web of Science and Embase. Third, we excluded studies of women undergoing ART procedures as low ovarian reserve is not the only reason for the use of these treatments. The main strength of this study is that it is one of the few systematic reviews to evaluate the influence of nutritional status on ovarian reserve in women of reproductive age.

## 5. Conclusions

Nutritional status can influence ovarian reserve in women of reproductive age. Overweight and obesity have a negative impact on ovarian function as women with a high BMI had significantly lower AMH levels and AFC than those with a normal BMI. Overweight and obesity can also affect oocyte quality, leading to higher rates of subfertility and infertility and a greater demand for ART procedures. Suboptimal nutritional status, however, can jeopardize the chances of ART success by inducing a detrimental inflammatory environment in the ovaries.

Future studies are needed to inform the design of prevention and health promotion strategies to improve the nutritional status of women of reproductive age.

## Figures and Tables

**Table 1:** Search strategy for PubMed.

Search Strategy
#1 (“ovarian reserve” [Title/Abstract] OR “ovarian reserve” [MeSH Terms])
#2 (“anti-mullerian” [Title/Abstract] OR “anti-mullerian” [MeSH Terms])
#3 1 AND 2
#4 (“nutritional status” [Title/Abstract] OR “nutritional status” [MeSH Terms])
#5 3 AND 4

**Table 2:** Quality of cross-sectional studies assessed using the AXIS critical appraisal tool.

Reference	1	2	3	4	5	6	7	8	9	10	11	12	13	14	15	16	17	18	19	20
Yang J et al., 2015 [27]	yes	yes	yes	yes	yes	yes	dnk	yes	yes	yes	yes	yes	dnk	dnk	dnk	yes	yes	yes	no	yes
Greenwood E et al., 2017 [28]	yes	yes	yes	yes	yes	yes	dnk	dnk	dnk	no	yes	yes	dnk	dnk	dnk	yes	yes	yes	dnk	yes
Moy V et al., 2015 [29]	yes	yes	dnk	yes	yes	yes	dnk	yes	yes	yes	yes	yes	dnk	dnk	dnk	yes	yes	yes	dnk	dnk
Giordano S et al., 2016 [30]	yes	yes	dnk	yes	yes	yes	dnk	yes	yes	yes	yes	yes	dnk	dnk	dnk	yes	yes	yes	no	yes
Bleil M et al., 2013 [31]	yes	yes	dnk	yes	yes	yes	dnk	yes	yes	yes	yes	yes	dnk	dnk	dnk	yes	yes	yes	dnk	yes
Feldman R et al., 2017 [32]	yes	yes	dnk	yes	yes	yes	dnk	yes	yes	yes	yes	yes	dnk	dnk	dnk	yes	yes	yes	dnk	yes
Phillips K et al., 2016 [33]	yes	yes	yes	yes	yes	yes	dnk	yes	yes	yes	yes	yes	dnk	dnk	dnk	yes	yes	yes	no	yes
Lin L et al., 2021 [34]	yes	yes	yes	yes	yes	no	yes	yes	yes	yes	yes	yes	dnk	dnk	dnk	yes	yes	yes	no	yes

1. Aims; 2. study design; 3. sample size justification; 4. target reference population; 5. sampling frame; 6. sample selection; 7. non-responders; 8. measurement validity and reliability; 9. risk factors and outcomes; 10. statistics; 11. overall methods; 12. basic data; 13. non-response bias; 14. non-responders; 15. internal consistency results; 16. comprehensive description results; 17. justified discussions and conclusions; 18. limitations; 19. conflict of interest; 20. ethical approval. DNK, Does not know; NR, no reply.

**Table 3:** Quality of cohort and case–control studies assessed using the Newcastle–Ottawa Scale.

Reference	1	2	3	4	5	6	7	8
Cohort studies								
Malhotra N et al., 2021 [35]	*	*	*	*	**	*	*	-
Berwanger da Silva A et al., 2013 [36]	*	*	*	*	**	*	*	*
Hvidman H et al., 2013 [37]	*	*	*	*	**	*	-	-
Lambert-Messerlian G et al., 2016 [38]	*	-	*	*	*	*	-	
Bragg J et al., 2012 [39]	*	*	*	*	*	*	-	-
Tabbalat A et al., 2017 [40]	*	*			*		*	*
Hardy T et al., 2018 [41]	-	*	*	*	**	*	*	*
Case-control studies								
Makolle S et al., 2021 [42]	-	*	-	*	*	-	*	-
Zhou S et al., 2021 [43]	-	*	-	*	**	*	*	*
Lefebvre T et al., 2017 [44]	*	*	*	*	**	*	-	-
Sahin A et al., 2017 [45]	-	*	-	*	*	*	*	*

1. Representativeness; 2. selection of non-exposed cohort; 3. ascertainment of exposure; 4. outcome; 5. comparability of cohorts; 6. assessment of outcome; 7. follow-up; 8. adequacy of follow-up. A maximum of one star is allocated for each domain within the “selection” and “outcome” categories, and a maximum of two stars is allocated for “comparability”. * or **; “ A study cab be awarded a maximim of one star for each numbered item within the selectionans exposure categories. A maximum of two stars can be givenfor comparability.

**Table 4:** PEDro Tool for randomized clinical trials.

Reference	1	2	3	4	5	6	7	8	9	10	11
Ganer H et al., 2017 [46]	YES	YES	YES	YES	YES	NO	NO	NO	YES	YES	YES

1. Eligibility criteria; 2. subjects were randomly allocated to groups; 3. allocation; 4. the groups were similar at baseline; 5. there was blinding of all subjects; 6. blinding of all therapists who administered the therapy; 7. there was blinding of all assessors who measured at least one key outcome; 8. measures of at least one key outcome were obtained from more than 85% of the subjects initially allocated to groups; 9. all subjects for whom outcome measures were available received the treatment or control condition as allocated or, where this was not the case, data for at least one key outcome were analyzed by “intention to treat”; 10. results between groups are reported for at least one key outcome; 11. the study provides both point measures and measures of variability for at least one key outcome.

**Table 5:** Study characteristics.

Authors, Year	Country	Year	Mean Age	Sample	Objective	Reported Strengths and Limitations	Study Design
Zhou S et al., 2021 [43]	China	2021	33.7	638	To explore differences in ovarian reserve between healthy fertile and infertile Chinese women of reproductive age	Influence of ethnicity not explored	Case-control
Philips K et al., 2016 [33]	Australia	2016	35	693	To determine whether women with BRCA1 or BRCA2 mutations have diminished ovarian reserve	AMH does not provide a direct measure of the primordial follicle pool; study design not suitable for assessing clinical implications of lower AMH concentrations observed among BRCA1 mutation carriers	Cross-sectional
Hardy T et al., 2018 [41]	Scotland	2018	33.9	69	To explore the ability of latent class analysis to identify subgroups based on cardiometabolic, psychological, and reproductive health parameters and describe AMH levels within these subgroups	Small sample composed of postpartum women with known fertility	Cohort
Sahin A et al., 2015 [45]	Turkey	2015	34.2	70	To compare ovarian reserve with AMH levels, AFC, and ovarian volume in women with Behçet disease and healthy women	Cross-sectional study primarily involving women with mucocutaneous manifestations and women previously treated with corticosteroids and azathioprine; results may differ for women who use cytotoxic agents (e.g., cyclophosphamide) and women with major organ involvement (neurologic major vessel involvement)	Cross-sectional
Lin L et al., 2021 [34]	China	2021	35.1	1935	To investigate the association between serum testosterone and AMH levels in infertile women	Retrospective, cross-sectional study; unable to draw conclusions on a causative link between serum testosterone levels and AMH; results not applicable to the general population as the sample only included infertile women	Cross-sectional
Makolle S et al., 2021 [42]	France	2021	29	82	To evaluate AMH levels in patients with functional hypothalamic anovulation	Retrospective study not representative of the general population	Case-control
Moy V et al., 2015 [29]	USA	2015	36	350	To determine the effects of obesity on AMH levels in women from different racial backgrounds	Strengths: inclusion of women from different racial backgrounds. Limitations: small sample with only women being evaluated for infertility	Cross-sectional
Giordano S et al., 2016 [30]	USA	2016	NR (18–45)	124	To determine whether BRCA1 mutations negatively influence ovarian reserve	Strengths: exclusion of women with BRCA2 mutations (other studies have not differentiated between BRCA1 and BRCA2)	Cross-sectional
Tabbalat A et al., 2017 [40]	UAE	2017	31.7	763	To explore potential differences in ART outcomes between Arabian Peninsula and Caucasian women	Strengths: inclusion of a homogeneous population from the Arabian Peninsula. Limitations: small sample.	Cohort
Ganer H et al., 2017 [46]	Israel	2017	35.6	46	To compare short-term ovarian reserve and operative complications in women who underwent salpingectomy vs. tubal ligation during cesarean section	Limitations: small sample and a lack of long-term follow-up. Strengths: randomized trial.	Clinical trial
Bleil M et al., 2013 [31]	USA	2013	35.2	951	To determine whether variability in reproductive aging is related to cardiovascular risk factors in the premenopausal period	Limitations: cross-sectional design	Cross-sectional
Feldman R et al., 2017 [32]	USA	2017	28.4	252	To determine the association between AMH levels and metabolic syndrome in young women with PCOS	Limitations: cross-sectional design	Cross-sectional
Malhotra N et al., 2021 [35]	India	2012	32.1	183	To determine whether increased BMI negatively affects ovarian reserve in infertile Asian women undergoing in vitro fertilization	Limitations: small number of overweight and obese women compared to normal-weight women	Cohort
Berwagner da Silva A et al., 2013 [36]	Brazil	2013	32.5	80	To investigate the influence of tubal ligation on ovarian reserve	Limitations: short follow-up (1 year); some patients lost to follow-up; no control group	Cohort
Hvidman H et al., 2016 [37]	Denmark	2016	33.1	632	To compare AMH levels and AFC between infertile women aged < 40 years and women in the same age group with no history of infertility	Strengths: small sample. Limitations: women were recruited for measurement of ovarian reserve at different time intervals.	Cohort
Lambert-Messerlain G et al., 2015 [38]	USA	2015	33.7	45	To determine, using the most advanced immunoassay technique available, whether AMH levels vary during the normal menstrual cycle.	Limitations: few smokers	Cohort
Bragg J et al., 2012 [39]	Philippines	2012	21.5	294	To determine whether ovarian reserve in early adulthood is related to measures of life history scheduling (age at menarche) and reproductive effort (parity)	Strengths: longitudinal (cohort) study and a large sample	Cohort
Lefebvre T et al., 2017 [44]	France	2017	28	691	To explore the effects of metabolic status on serum AMH levels in women with and without PCOS	Strength: large sample	Case-control
Yang J et al., 2015 [21]	Taiwan	2015	25	186	To investigate associations between iron levels, obesity, and ovarian reserve in women with PCOS	Limitations: small control group, young age (<30 years old), and lack of control group of obese women.	Cross-sectional
Greenwood E et al., 2017 [28]	USA	2017	32.7	503	To determine whether women with idiopathic infertility have a lower ovarian reserve than healthy controls not seeking fertility treatment	Limitations: retrospective study	Cross-sectional

AFC, Antral follicle count; AMH, anti-Müllerian hormone; BMI, body mass index; E2, estradiol; FSH, follicle-stimulating hormone; HDL, high-density lipoprotein; LH, luteinizing hormone; PCOS, polycystic ovary syndrome; UAE, United Arab Emirates; USA, United States of America.

**Table 6:** Summary of variables used to assess nutritional status and ovarian reserve.

Study, Authors, Year	Subgroups	Total Sample, *n*	Nutritional Status Variables	Ovarian Reserve Variables	Other Variables
Zhou et al., 2021 [43]	Fertile and infertile women	638	Weight, height, BMI	FSH, LH, FSH:LH ratio, E2, AMH, AFC	-
Philips K et al., 2016 [33]	BRCA1 and BRAC2 mutation carriers and non-carriers	693	Weight, height, BMI	AMH	Toxic habits: smoking Obstetric parameters: parity, age at first delivery
Hardy T et al., 2018 [41]	Three classes (subgroups) based on cardiometabolic, psychological, and reproductive factors	69	BMI	FSH, LH, E2, AMH	Biochemical parameters: cholesterol, HDL, triglycerides, glucose
Sahin A et al., 2015 [45]	Women with and without Behçet disease	70	Weight, height, BMI	AMH, FSH, LH, E2, AFC, ovarian volume	-
Lin L et al., 2021 [34]	Four groups of women with different serum testosterone levels	1935	Weight, BMI	AMH, FSH, LH, E2	Biochemical parameters: vitamin D, prolactin
Makolle S et al., 2021 [42]	Women with functional hypothalamic anovulation and controls	82	BMI	FSH, LH, AMH, AFC	Biochemical parameters: androstenedione, total testosterone, prolactin
Moy V et al., 2015 [29]	African American, Asian, Caucasian, and Hispanic women	350	BMI	AMH, FSH, AFC	Toxic habits: smoking Obstetric parameters: parity, age at first delivery
Giordano S et al., 2016 [30]	*BRCA*-positive and *BRCA*-negative women	145	BMI	AMH	Toxic habits: smoking Obstetric parameters: parity Others: tamoxifen use
Tabbalat A et al., 2017 [40]	Arabian Peninsula and Caucasian women undergoing ART procedures	763	BMI	FSH, AMH, AFC	Ovarian stimulation parameters: total duration, gonadotropin dosage, estrogen, mature oocytes
Ganer H et al., 2017 [46]	Women undergoing tubal ligation vs. bilateral salpingectomy during cesarean section	46	BMI	AMH	Biochemical parameters: postoperative hemoglobin
Bleil M et al., 2013 [31]	Healthy women (cyclists)	951	Waist circumference	AMH	Biochemical parameters: triglycerides, LDL, insulin resistance
Feldman R et al., 2017 [32]	Women with PCOS	252	Weight, height, BMI	AMH	Biochemical parameters: total cholesterol, triglycerides, HDL, LDL, glucose, insulin, TSH, prolactin, DHEAS, 17-OH progesterone
Malhotra N et al., 2013 [35]	Women from an infertility clinic divided into three groups based on BMI (normal weight, overweight, and obese)	183	BMI	AFC, ovarian volume, inhibin B, FSH, LH	-
Berwagner da Silva, A. et al., 2013 [36]	Women undergoing tubal ligation	80	BMI	AMH and AFC Toxic habits: smoking Others: surgical technique	-
Hvidman H et al., 2016 [37]	Women with and without a history of infertility	632	BMI	AFC, ovarian volume, AMH, FSH, LH	-
Bragg J et al., 2012 [39]	Non-pregnant women	294	BMI	AMH	Toxic habits: smoking Gynecologic/obstetric parameters: age at menarche, parity
Lefebvre T et al., 2017 [44]	Women with and without PCOS	691	BMI	AFC, FSH, LH	Biochemical parameters: DHEAs, 17-OH progesterone, SHGB
Yang J et al., 2015 [27]	Obese and non-obese women with and without PCOS	186	BMI	Ovary size, AMH, AFC	-
Greenwood E et al., 2017 [28]	Women with and without idiopathic infertility	503	BMI	FSH, LH, AFC	-

BMI: Body mass index; FSH: follicle-stimulating hormone; AMH: anti-Müllerian hormone; HDL: high-density lipoprotein cholesterol; Hb: haemoglobin; LH: luteinising hormone; E2: estradiol; AFC: antral follicle count; PRL: prolactin; LDL: low-density lipoprotein cholesterol; TSH: thyroid-stimulating hormone; DHEAS: dehydroepiandrosterone sulphate; 17OHP: 17-hydroxyprogesterone; SHGB: sex-hormone-binding globulin.

**Table 7:** Summary of the relationship between BMI and ovarian reserve and study results and conclusions.

Authors, Year	Relationship between BMI and Ovarian Reserve	Results	Conclusions
Zhou et al., 2021 [43]	BMI was included as a confounder.	Differences between cases and controls for AFC, AMH, and ORPI (*p* < 0.01). In both groups, these variables decreased with increasing age. Positive correlation between AMH and AFC (*p* < 0.001) and negative correlation between age and AFC, AMH, and ORPI (*p* < 0.05). Significant differences in age (*p* < 0.001), E2 (*p* < 0.01), and AMH (*p* < 0.01) between cases and controls. After controlling for confounding factors (age, BMI, total testosterone, and LH), no differences were observed for AMH, FSH, E2, or AFC (*p* < 0.05).	Diminished ovarian reserve is a manifestation of aging and is influenced by several factors. No differences were observed for ovarian reserve between fertile and infertile women when adjusting for confounders, and there was no correlation between ovarian reserve and infertility.
Philips K et al., 2016 [33]	BMI was included as a confounder.	AMH was negatively associated with age (*p* < 0.001). BRCA1 and BRCA2 carriers were younger than non-carriers when blood was drawn (*p* ≤ 0.031). BRCA1 carriers had on average 25% (95% CI: 5–41%, *p* = 0.02) lower AMH concentrations than non-carriers and were more likely to have AMH concentrations in the lowest quartile for their age (OR, 1.84; 95% CI, 1.11–303; *p* = 0.02). No evidence was found for an association between AMH concentrations and presence of a BRCA2 mutation (*p* = 0.94).	BRCA1 mutation carriers had on average 25% lower AMH concentrations than non-carriers.
Hardy T et al., 2018 [41]	There are differences in AMH levels between lean (3.19 ±2.81 ng/mL) and obese (2.3 ± 2.0 ng/mL) women, but there are no statistically significant differences (*p* = 0.143)	Latent class analysis was used to classify people based on cardiometabolic, psychological, and reproductive factors. Three classes (subgroups) were identified. Class 1 had the highest mean AMH levels and the lowest mean cholesterol levels. Class 3 had the lowest mean AMH levels and the highest mean cholesterol and triglyceride levels.	Low ovarian reserve was correlated with cardiovascular and psychological factors.
Sahin A et al., 2015 [45]	BMI is not associated with ovarian reserve.	No statical differences were observed between women with Behçet disease and healthy women for mean age, deliveries, miscarriages, live births, BMI, FSH, LH, E2, prolactin, ovarian volume, or AFC (*p* > 0.05). Differences for AMH levels were also non-significant (*p* = 0.468). There are no significant correlations between AMH levels and age, BMI, FSH, LH, E2, prolactin, AFC, ovarian volume (*p* > 0.025) in women with Behçet disease or healthy women.	Ovarian reserve appeared to be preserved in women with Behçet disease. AMH levels were similar in women with Behçet disease and healthy women.
Lin L et al., 2021 [34]	BMI is more closely associated with testosterone levels and higher levels of testosterone and AMH.	Women in the lowest quartile (Q1, low testosterone) had significantly lower AMH levels than those in the top quartile (Q4, high testosterone) (*p* < 0.001). After controlling for age, bodyweight, BMI, and FSH, higher testosterone quartile categories were associated with higher AMH levels. Binary logistic regression analyses showed an 11.44-fold increase in the chances of diminished reserve in Q1 vs. Q4 and a 10.41-fold increase in the chances of excess ovarian reserve in Q4 vs. Q1 (*p* < 0.001).	Serum testosterone levels were positively associated with AMH levels, suggesting that androgen insufficiency is a potential risk factor for diminished ovarian reserve.
Makolle S et al., 2021 [42]	Positive correlation between BMI and LH levels in women with FHA and PCOM. No other influence observed for BMI.	Overall, 46.7% of women with FHA had PCOM. When these patients were excluded, AMH levels were significantly lower in women with FHA than in controls (*p* < 0.002). In the group of women with FHA, those with PCOM had significantly higher AMH and BMI levels than those without PCOM. Women with PCOM had significantly lower LH, FSH, and androstenedione levels than controls (*p* < 0.0001, *p* < 0.002, and *p* < 0.05, respectively). A significant positive correlation was observed between AMH and LH levels in controls but not in women with FHA.	AMH levels were not decreased in women with FHA, but when those with PCOM were excluded, the levels were significantly lower than in controls, supporting findings for other situations with gonadotropin insufficiency.
Moy V et al., 2015 [29]	There was a negative correlation between a high BMI and AMH levels in Caucasian women.	Age was negatively correlated with AMH and AFC in women from all racial backgrounds (*p* < 0.05). After controlling for age, PCOS, and smoking, a high BMI was negatively correlated with AMH in Caucasian women (*p* = 0.01).	A high BMI was negatively correlated with AMH in Caucasian women.
Giordano S et al., 2016 [30]	BMI was included as a confounder and does not appear to correlate with AMH levels.	BRCA1-positive women experienced a significant decline in AMH with age (*p* = 0.0011). BRCA1 mutation carriers aged > 35 years had lower AMH levels (<0.5 ng/mL) than younger women. After controlling for BMI, birth control duration, smoking, pregnancy, parity, and age > 35 years, BRCA1 was still strongly associated with low AMH levels (*p* = 0.037).	BRCA1-positive women aged > 35 years had lower AMH levels and therefore lower ovarian reserve than BRCA1-negative women.
Tabbalat A et al., 2017 [40]	BMI was included as a confounder and does not appear to correlate with AMH levels.	The women from the Arabian Peninsula had higher FSH levels (5.7 ± 2.5 vs. 4. 9 ± 2.8, *p* = 0.001) and lower AFC (13.9 ± 4.7 vs. 16.5 ± 4.3, *p* < 0.001) than Caucasian women. Fewer mature oocytes were retrieved from women from the Arabian Peninsula (15.6 ± 6.8 vs. 14.1 ± 8.4, *p* = 0.01), even though they required higher doses of gonadotropin. Women from the Arabian Peninsula had 2.5 (95% CI 2.1–3.9) fewer mature oocytes, even after controlling for confounding factors. A subanalysis within this cohort showed that Qatari women had a higher yield of mature oocytes than Emirati, Kuwaiti, or Saudi women. There were no differences in implantation, clinical pregnancy, or live birth rates when comparing women from the different countries in the Arabian Peninsula with each other or with Caucasian women.	Ethnic background was associated with low ovarian reserve and low ovarian response parameters in women undergoing their first cycle of intracytoplasmic sperm injection–embryo transfer.
Ganer H et al., 2017 [46]	BMI was included as a confounder, and no differences were observed between the two study arms (bilateral salpingectomy vs. tubal ligation during cesarean section).	The salpingectomy group was slightly older than the tubal ligation group (37.0 vs. 34.3 *p* = 0.02). There were no differences for parity, BMI, gestational age, or for AMH levels during pregnancy and postpartum. The mean increase in AMH was 0.58 ± 0.98 ng/mL in the salpingectomy group and 0.39 ± 0.41 in the tubal ligation group (*p* = 0.45). Cesarean sections with salpingectomy lasted on average 13 min longer (66.0 ± 20.5 vs. 52.3 ± 15.8 min, *p* = 0.01). No differences were observed in surgical complications or postoperative hemoglobin between the groups.	Salpingectomy is as safe an option as tubal ligation and, in addition, reduces the risk of ovarian cancer.
Bleil M et al., 2013 [31]	AMH is associated with a healthy cardiometabolic profile. Low and medium AMH levels were associated with a larger waist circumference and higher cholesterol levels. More longitudinal studies are needed to determine whether the association with a healthy cardiometabolic profile is mediated by BMI.	In the age-adjusted models, low (vs. high) AMH levels were associated with a 52.1% increase in the number of cardiometabolic risk factors. The increase in the number of cardiometabolic risk factors for medium vs. high levels was 46.0%. Low and medium (vs. high) AMH levels were associated with an increased risk of HDL (OR 1.81, *p* < 0.01 and OR 1.56, *p* < 0.05, respectively), waist circumference (2.01 and 1.88; *p* < 0.001), and hypertension (OR 2.37, *p* < 0.01 and OR 2.05, *p* < 0.1, respectively). The associations were weaker when BMI was included as a covariate (*p* > 0.05).	A higher ovarian reserve was associated with a healthier cardiometabolic risk factor profile.
Feldman R et al., 2017 [32]	AMH is positively correlated with SHBG and HDL cholesterol and negatively correlated with glucose, insulin, BMI, and blood pressure.	Median AMH was 5.1 ng/mL, and 23.8% of women had metabolic syndrome. A single unit decrease in AMH was associated with an 11% increase in the odds of metabolic syndrome (OR 1.11; *p* = 0.01). The strength of this association was maintained in the multivariate model (OR 1.09; *p* = 0.02) after adjusting for age and race. Women with AMH levels in the bottom tertile were twice as likely as those in the top tertile to have metabolic syndrome (adjusted OR, 2.1; 95% CI, 1.01–4.3). Total testosterone was not associated with metabolic syndrome or any of its components.	Low AMH levels predicted an increased risk of metabolic syndrome in young women with PCOS. The role of AMH in cardiometabolic risk stratification in obese women with PCOS needs to be clarified in longitudinal studies and in perimenopausal women.
Malhotra N et al., 2021 [35]	Overweight and obesity are correlated with low AFC and low inhibin B levels.	Age was comparable in obese, overweight, and normal-weight women. Mean duration of infertility was 8.38 years. Compared to normal-weight women, overweight and obese women had significantly lower inhibin B levels (*p* < 0.0259). Differences in AFC were not significant between the groups. Overweight and obese women, however, had a significantly lower AFC on the right side.	Overweight and obesity correlate with a low AFC and low inhibin B levels.
Berwagner da Silva A et al., 2013 [36]	AMH was associated with AFC on comparing women with a BMI < 25 and a BMI of 25–30; the association was not observed in women with a BMI > 30.	Fifty-two patients completed the study protocol. Median AMH was 1.43 ng/mL (IQR, 0.63–2.62) preoperatively and 1.30 ng/mL (IQR, 0.53–2.85) at 12 months (*p* = 0.23). Mean AFC was 8.0 (IQR, 5.0–14.0) before tubal ligation and 11.0 (IQR, 7.0–15.0) afterwards (*p* = 0.12). Increased postoperative AMH levels were associated with hormonal contraceptive use prior to tubal ligation.	Tubal ligation did not affect or induce changes in ovarian reserve. AMH was associated with AFC when women with a BMI < 25 and 25–30 were compared.
Hvidman H et al., 2016 [37]	Infertile women and women without a history of infertility had the same BMI, indicating an absence of association with AMH.	Infertile women had similar AMH levels (11%, 95% CI, 21–24%) and AFC (1%, 95% CI, 7–8%) to controls without a history of infertility in the age-adjusted linear regression analysis. The prevalence of very low AMH levels (<5 pmol/L) was similar in both groups (age-adjusted OR, 0.9; *p* < 0.001). Similar findings were observed after adjusting for smoking, BMI, gestational age at birth, previous conception, and chronic disease in addition to age.	AMH was not associated with BMI as infertile women and controls without a history of infertility had the same BMI.
Lambert-Messerlain G et al., 2015 [38]	BMI does not correlate with AMH.	Serum AMH levels varied significantly during normal menstrual cycles and peaked in the follicular phase. In the age-stratified analysis, variations in AMH levels during the normal menstrual cycle were significant only for women > 30 years.	BMI and smoking were not correlated with AMH.
Bragg J et al., 2012 [39]	BMI is not associated with AMH, but it was studied as a confounder.	Mean AMH was 4.3 ng/mL. In the multiple regression models, women who experienced menarche earlier had significantly higher AMH levels in their young adult lives (*p* < 0.05). Women with two (*p* < 0.05) and three or more (*p* < 0.01) children had significantly lower AMH levels than those without children. These associations were independent of age, smoking, and BMI.	Individual variations in life history scheduling and reproductive history might contribute to variations in ovarian reserve. They also demonstrate the usefulness of AMH as a tool for reproductive ecology.
Lefebvre T et al., 2017 [44]	AMH correlates with BMI ≥ 25 in women with PCOS, with lower mean levels.	Mean serum AMH levels were slightly, and not significantly, lower in overweight and obese women with PCOS than in normal-weight women with PCOS (*p* < 0.05). BMI and AMH were not correlated in the control group after bivariate analysis. In the PCOS group, the correlation was significant (*p* = 0.0001) but weak (r = −0.177). Stepwise multiple regression analysis yielded a significant model, with AFC, serum androstenedione, BMI, serum LH, and FSH accounting for 38.6%, 3.4%, 1.4%, 0.7% and 1.4% of total serum AMH variability, respectively.	In women with PCOS, AMH levels were significantly correlated with BMI, insulin levels, hip circumference, and levels of FSH, LH, estrogen, and testosterone.
Yang J et al., 2015 [27]	Obese women with PCOS have lower AMH levels than non-obese women with PCOS. In non-obese women with PCOS, the AMH levels were 64 pM (8.96 ng/mL) vs. 37 pM (5.18 ng/mL) in obese women with PCOS. Obesity was associated with diminished ovarian reserve and reduced menstrual period frequency (*p* < 0.0001).	Ferritin and transferrin-bound iron levels were significantly higher in women with PCOS than in healthy normal-weight controls. Obese women with PCOS had higher ferritin levels (*p* = 0.006) and lower AMH levels (*p* < 0.0001) than non-obese women with PCOS. In the univariate analysis, AMH levels and mean ovarian volume were inversely related to ferritin levels, HOMA-IR, and BMI in women with PCOS. After controlling for confounders, BMI and ferritin levels were significantly correlated with lower AMH levels and reduced ovarian volume, respectively.	Obese women with PCOS had higher iron levels but lower AMH levels than non-obese women with PCOS. Increased iron levels and obesity appear to be related to insulin resistance, metabolic disorders, decreased ovarian reserve, and reduced menstrual period frequency.
Greenwood E et al., 2017 [28]	Infertile women have a larger waist circumference and are more likely to have a history of smoking.	AMH, AFC, and AMH/AFC ovarian reserve indices did not differ between infertile women and controls after adjusting for age, race, smoking history, and study site.	AMH, AFC, and AMH/AFC ovarian reserve indices did not differ between women with idiopathic infertility and healthy controls not seeking fertility treatment after controlling for age, race, BMI, and smoking. Infertile women, however, had a larger waist circumference and were more likely to have a smoking history.

AFC, Antral follicle count; AMH, anti-Müllerian hormone; BMI, body mass index; DHEAS, dehydroepiandrosterone sulfate; E2, estradiol; FSH, follicle-stimulating hormone; HDL, high-density lipoprotein; HOMA-IR, homeostatic model assessment for insulin resistance; IQR, interquartile range; LH, luteinizing hormone; ORPI, ovarian response prediction index; PCOS, polycystic ovary syndrome; SHGB, sex-hormone-binding globulin; UAE, United Arab Emirates; TL, tubal ligation; PCOM, polycystic ovarian morphology; FHA, functional hypothalamic anovulation.

**Table 8:** Results according to the influence of BMI on AMH and AFC.

Authors, Year	←↑ BMI		↓ BMI	
	AMH	AFC	AMH	AFC
Zhou S et al., 2021 [43]	-	-	-	-
Philips K et al., 2016 [33]	-	-	-	-
Hardy T et al., 2018 [41]	-	-	-	-
Sahin A et al., 2015 [45]	-	-	-	-
Lin L et al., 2021 [34]	←-	-	-	-
Makolle S et al., 2021 [42]	↑ In women with PCOM		-	-
Moy V et al., 2015 [29]	¯	-	-	-
Giordano S et al., 2016 [30]	-	-	-	-
Tabbalat A et al., 2017 [40]	-	-	-	-
Ganer H et al., 2017 [46]	-	-	-	-
Bleil M et al., 2013 [31]	↑← AMH healthier cardiometabolic profile	-	-	-
Feldman R et al., 2017 [32]	↓	-	-	-
Malhotra N et al., 2021 [35]	-	↓	-	-
Berwagner da Silva A et al., 2013 [35]	-	BMI was associated with AFC in a comparison of women with a BMI < 25 and a BMI of 25–30	-	-
Hvidman H et al., 2016 [36]	-	-	-	-
Lambert-Messerlain G et al., 2015 [37]	-	-	-	-
Bragg J et al., 2012 [38]	-	-	-	-
Lefebvre T et al., 2017 [43]	↓	-	-	-
Yang J et al., 2015 [20]	↓	-	-	-
Greenwood E et al., 2017 [27]	↓	-	-	-

**←↑**: increase; **↓**: decrease; -: no relationship; BMI: body mass index; AMH: anti-Müllerian hormone; AFC: antral follicle count; PCOS: polycystic ovary syndrome; PCOM: polycystic ovarian morphology.

## Data Availability

All data can be found in the article.
